# Progress and Practice

**Published:** 1898-12

**Authors:** Robert Roxburgh

**Affiliations:** Physician to the Weston-Super-Mare Hospital and Dispensary; Consulting Physician to the Royal West of England Sanatorium


### The Bristol

### Medico-Chirurgical Journal.

DECEMBER, 1898.

### PROGRESS AND PRACTICE.

The Presidential Address, delivered on October 12th, 1898, at the Opening of the Twenty-fifth Session of the Bristol Medico-Chirurgical Society.

BY

ROBERT ROXBURGH, M.B. Ed., F.R.C.S. Ed.,

*Physician to the Weston-super-Mare Hospital and Dispensary; Consulting Physician to the Royal West of England Sanatorium.*

In occupying the presidential chair of this learned Society, my first duty is to express a very real sense of gratitude to the members for their kindness in electing one of their members as President who is non-resident in this city, and who, alas, has small claim to compete in distinction and ability with many of the brilliant men who in the past have guided the deliberations of the Bristol Medico-Chirurgical Society. I accept this honour not as an individual, but rather as the representative of those provincial members who in increasing number have learned to appreciate the instructive papers and discussions which make the evenings spent in this room so pleasant and profitable. It is with peculiar pleasure also that I take this opportunity of acknowledging the invariable hospitality of the members of the profession in Clifton and Bristol to those who come from a distance, and who would fain, if they could, reciprocate some of the courtesy so constantly extended to them here.

My second duty, a less easy and enjoyable one than the acknowledgment of kindness, is to inaugurate the work of our new Session with an address which shall be, if not interesting, at least instructive.

In selecting a subject on which to address you I have realised more than ever the enormous, the limitless field, historic, scientific, practical, and academic, which is covered by modern medicine, and the exceeding difficulty of choice amid such an *embarras de richesse.* Never before has medicine presented such a prodigality of captivating topics to the observer and the thinker as at this moment. What amazing progress the Victorian age has witnessed in biological discovery, and in the revelation of rational and demonstrable bases for treatment, instead of the gropings of empiricism and of purely speculative theory! Not to speak of the cellular pathology which is the very A B C of our present knowledge, and with which the immortal name of Virchow is associated, and to come to more immediately recent times—it is a high privilege to have lived through an era which has seen the practical abolition from surgical practice of acute suppurative inflammation, pyæmia, and septicæmia, and has widened the scope of surgical procedure beyond the wildest dreams of preceding generations; in which it has become as safe to open the peritoneal or the synovial cavities as to amputate a finger; an era in which the localisation of cerebral functions has been demonstrated, and it has become possible to cure a paralysed epileptic lunatic by a simple operation on the cerebral cortex.

I believe the rising generation of young surgeons can have no adequate sense of the far-reaching results of Lister's splendid work. They have not seen, nor smelt, as I have, a large lacerated wound of the hand treated in a great city hospital by linseed poulticing! I can remember as if it were yesterday the sickening odours of the surgical wards of a particular Royal Infirmary, where acute surgical emphysema, erysipelas, and suppuration following operations were quite as much the rule as the exception, and were regarded as inevitable. Compare such a state of things with what we see to-day—the painlessness of surgical cases, the absence of fever, the approximation in fact of all operative cases to the conditions which hold with an unbroken skin, and remember that the man to whom the world owes one of its mightiest debts had for years to fight an uphill battle absolutely alone, to be called a faddist, and to have his earlier methods of campaign decried and ridiculed, though he was ever the first to discard any part of his armature which was proved to be unnecessary.

It is well also to remember what light was shed on the processes of repair and on the functions of the leucocyte by antisepsis. One of the most fascinating of the problems now engaging, in laboratory and sick-room, many of the master-minds of the profession is the part played by the leucocyte in the economy, and the discoveries of Cohnheim, Recklinghausen, and Metchnikoff form a chapter of romantic interest; but some of these were foreshadowed by what was first witnessed under aseptic conditions—the healing of deep wounds by the organisation of blood-clot, without a trace of pus, and the absorption of catgut and other organic substances introduced into aseptic tissues.

It would be absurd to deny that magnificent results in surgical cases were often obtained in the pre-Listerian days. Happily, owing to the antiseptic properties of the healthy tissues, union by first intention was common enough. But there was no *certainty* of a favourable issue, while wounds already septic too often ended in death. What a picture Sir William Mac Cormac drew[1] the other day of the awful destruction of life among the French wounded in the war of 1870!

The science of bacteriology was only in its infancy when I was a medical student, and the idea of *pathogenic* organisms was just beginning to enter the minds of pathologists as a remote possibility. Who could then have believed that traumatic tetanus, at that time said to be caused by venous engorgement of the spinal cord, would in a few years be proved to be due to

[1] For this see the present number of this *Journal*, p. 301.

an organism flourishing in dirt and garden soil? The new knowledge is proving many diseases to be of toxic origin which were formerly attributed to "congestion" or some equally insufficient cause. I well remember the dissatisfaction with which, as a student, I heard our Professors talk of the "essential paralysis of children," a name well designed to cover the ignorance then existing of the actual morbid process involved, and now familiar to us as "anterior poliomyelitis." Who can doubt that the real "essential" of this disease is a poison attacking the motor cells of the anterior columns with a certainty of selection no less specific than the selective affinities of the diphtheria poison, or those of lead and arsenic?[1] Had I been able to follow my inclination, I should have addressed myself to-night to the subject of *immunity*, which is gradually undergoing illumination, and seems to be on the verge of complete exposition, though the key which is to unlock some of its principal difficulties has yet to be found. But circumstances are tyrannical; and as I saw that I could not give an adequate account of what has been done up to the present in clearing up its difficulties, I abandoned it in favour of a less ambitious theme. The members of this Society are not pathologists only, but practitioners; and if I venture to-night to leave the many questions of modern pathology alone, and to deal with some of the *general conditions* which affect us as practitioners, I hope I shall not employ your time quite uselessly.

It is hardly possible to be eighteen years in practice without receiving and perhaps giving a few hard rubs, without having painful misgivings at times on comparing our youthful aspirations with the measure and kind of our success, without seeing in the relationships between the profession and the public, as well as in those of the profession itself, facts that disappoint us and evils that call for remedy. Most of us have doubtless entered the medical profession with minds elated with enthusiasm. Coming directly from some great medical school, where we have enjoyed constant and stimulating intercourse

[1] Taken from the *Australasian Medical Gazette* of April 24, 1897, there is in the *Medical Annual* for this year an interesting reference to an epidemic of anterior poliomyelitis due to phosphorus poisoning.

with men of the front rank, have got glimpses of many untrodden paths inviting us to discovery, have seen medical knowledge daily emerging from empiricism and entering the region of exact science, have heard on all hands suggestions of possible future harmony between apparently contradictory facts, and have seen the application of pathology, therapeutics, and new surgical methods to large numbers of deeply interesting cases, we have felt that there was no profession in the world to compare with medicine. It might sometimes occur to us that it would have to be in the future a means of livelihood as well as the occasion of all-absorbing study; but what were monetary considerations in comparison with that union of scientific and human interest which charmed us like a spell and made our work a perpetual delight? It was not money we wanted, but good clinical cases, and opportunities for such thought and research as we should put at their disposal and be the means of their cure. True, if we were Hospital Residents, we might get a very occasional fee for some medico-legal service such as an inquest. Such a fee had a preciousness all its own. It marked a climax in our career, the turning-point at which we should no longer pay but receive money, and it brought with it a new consciousness of pride. We were in a position to earn our living, and could henceforth afford to look with a kindly patronage on our juniors and inferiors who were only beginning their studies. What a difference five years had made on us! How much we knew, how sure were our diagnoses! Then what genial *camaraderie* we enjoyed in hospital life! How interesting life was! We knew nothing of drudgery. Work in the wards was laborious, but it had its humours, and every case had something to teach us. Moreover, all the heavier responsibilities were borne by our chiefs; while in the society of our fellow-graduates and in the frequent advent of casualties, poisonings, cases of asphyxia, or other exciting episodes, we were drawn together in various ways, and every day had its new and instructive experiences.

But when we embarked on private practice, and donned the frock-coat and tall hat which are the practitioner's official uniform, we found matters very different from what we had expected. If we received a midnight call, it was too often to some perfectly trivial case which we felt might very properly have waited till morning. The precious hours which we had decided to devote to microscopical research were constantly interfered with. No sooner had we got out our apparatus than we were sent for to see some one living a mile or two away who had had a severe stomach-ache, but was "better now," as we were told the moment we reached the house. The special courses of study we had prescribed for ourselves were perpetually interrupted, till continuous thought became almost an impossibility, and we had to learn a new and difficult art, an art which many never succeed in mastering—the art of *detachment*, of passing in a moment from one train of thought to another, and another, and another, without losing the impressions of any. Then, more difficult still, came the art of keeping our temper, of looking placid and bland while inwardly raving! Gradually there dawned on our consciousness the painful truth that *personality* is one of the most important factors of success in private practice. A man may be professionally and morally equipped with the highest qualifications: he may be of sound judgment, good memory, observant, and admirably informed, with a loyal and kind heart; but if he has not "a good bedside manner" (detestable phrase), let him make up his mind to be either unsuccessful or perilously slow in forming a *clientèle*. A friend once said to me on returning from his holiday: "Oh, it's the *smiling* that I abhor; to have to sit and smile when I don't feel inclined makes me sick." Certain personal peculiarities are favourable to success. A man of conspicuous ugliness is sometimes considered "very clever," and is much sought after. But a "bedside manner," which implies both deep sympathy with the sufferer in the extreme seriousness of his case and absolute omniscience in regard to its nature and proper treatment,—this is what the public often demand, and the demand creates the supply. Facts like these and other experiences gradually may have wrought in us the conviction that the ambitions which were the inspiration of our earlier years were inconsistent with the actual conditions of ordinary provincial medical practice.

But if I have been echoing the feelings of any one now present, and if it be thought that my remarks are too pessimistic, let me at once say that there is another side to the picture. The great mass of men are not made for distinction or mighty achievement, but for faithfulness in the exercise of a limited amount of ability, and devotion, even under severe restrictions, to thoroughly useful ends. The teaching of Browning is a welcome gospel to the man of but one or at most two talents, pronouncing as he does with joyful emphasis that the real value of a life, so far as the individual is concerned, is not to be found in the magnitude of its tangible results but in the actual struggle and effort which true work implies. There are many rewards in our daily work. The writing of prescriptions may be a monotonous task, but at longer or shorter intervals we are brought face to face with some case of profound interest, and which tests our knowledge and skill to the uttermost. Our patients as a rule are both reasonable and grateful, far more so at times than we feel we deserve, and our converse with intelligent minds is often most enjoyable. Then have we not the satisfaction of knowing that we belong to (with perhaps one exception) the least sordid and most unselfish of all the professions, and that we can often fill the office of a friend and counsellor in matters extra-medical and of extreme importance?

Now, it is as practitioners of the *art* of healing, as apart from its abstract and scientific aspects, that the *commercial* element first enters into our lives. Though it is the glory of medicine that its primary object is not money-getting, but the benefit of humanity, yet we are all agreed that the labourer is worthy of his hire, and so far from ignoring his fees every sensible man will see their fitness and insist on their payment. Unfortunately, however, it is from this side that breaches of ethical rule are apt to occur. The man who spends his days in a laboratory has few temptations to personal aggrandisement, but he whose income depends on the number of his patients is in a different position. Let me here, in the seat of a great medical school, assert that the principles of medical ethics are not taught to senior students as they should be, and students leave their school often without ever having heard the subject mentioned. It is frequently said that medical ethics are comprised in the saying: "Do to others as you would be done by;" but that is not strictly true, for the honour of the profession as a whole, and not merely our individual advantage, is concerned in the maintenance of a high ethical standard, and perfectly upright men may make grave mistakes of etiquette without a suspicion that they are compromising principle. I have known a high-toned young practitioner accept the post of medical officer to a notorious medical aid association, and who, the moment its true character was pointed out to him, resigned his office. I have known a man fresh from laboratory work abroad, acting on the advice of lay friends, advertise in a newspaper the fact that he had purchased a certain practice, totally unconscious meanwhile that he was committing an unprofessional act. Many other instances might be named where ignorance and inexperience have had very unpleasant consequences—strained relationships perhaps, or open rupture, between doctors practising in the same vicinity, and who ought to have been on the friendliest terms. Of course there are other cases where unscrupulous men, or men without gentlemanly instincts, consciously and wilfully advertise themselves, or push their practice in underhand ways. For such the penalty must be isolation from the medical brotherhood of a district.

It is much to be desired that before leaving college all senior students should be fully instructed in this important matter, and have definite illustrations put before them of what the higher professional opinion requires. The responsibility rests with teachers. Students are at a most impressionable age, and from the men that they revere are most ready to learn. I have never forgotten the words which I once heard Lister address to his class: "The worst forms of quackery are those practised within the ranks of the profession," and it is absurd to assume that that truth does not require reiteration in the ears of each succeeding generation.

The crowded condition of the medical profession renders competition and the struggle for existence greater year by year. In 1881 there were 23,275 medical men on the register of the

United Kingdom, that is one to 1514 of the population. In 1891 the proportion was one to 1289 of the population. In these ten years the population had increased by 2,862,493, and the registered doctors by 6,280, *i.e.* one to every 456 of the population. The number on the register is now (December 31st, 1897) 34,642. As there has been no census since 1891, it is impossible to state the present proportion with exactness, but the analogy of the previous years shows that a proportional increase still continues.

This competition is of course much greater in towns than in the country, and while the average income of the country doctor is far below what it ought to be, the inequality of income is greatest in the large centres. This is partly owing to the growth of specialism. A case which can be admirably dealt with by a well-educated general practitioner is taken out of his hands, as the patient insists on having a specialist, though he has to pay five or ten times as much for that privilege. The public has its medical fetiches, and to many the words "Harley Street" have an almost sacred sound, and a "London opinion" is considered to have a priceless value. Let me not be understood as minimising the importance and absolute necessity of specialism. I merely say that much of the highly remunerative work of the specialist could be done no less efficiently by the general practitioner. It is sometimes a good thing for one of the latter who may have enjoyed special facilities for its study to adopt a speciality in addition to his general work. If he meets a case beyond his powers he will readily recognise this and will call in an exclusive specialist.

Before passing from this topic of the specialist consultant, let me refer to a practice most excellent in its purpose, but liable to abuse. *Diet* has in our time become a much more important item in treatment than it used to be. The physiology of digestion and metabolism is better understood than ever before, and it is hopeless to treat most diseases without careful attention to its laws. But the plan of tabulating, not only the ingredients, but the very quantities of a patient's diet is often overdone, and is not unfrequently adopted in quite unsuitable cases. I believe that the minute directions sometimes given to patients have the opposite effect from what is intended. They may relieve immediate symptoms, but they manufacture hypochondriacs, a class already much too numerous. A gentleman known to me, who had never had a doctor in his life, had occasion to consult an eminent specialist. Few vegetables were admitted in his dietary, but amongst these a leading one was "flageolets." That gentleman came away from the consulting-room with a new conception of the importance of his case. A man whose only vegetable was flageolets must be a man out of the common. Covent Garden Market was consequently routed for the mysterious vegetable, and doubtless it was eaten with a solemn gusto. Many patients are only too ready to concentrate their thoughts on their interiors, and our hygienic rules for such should be accompanied by a distinct understanding that they are essentially temporary, and by the recollection that to turn a person's attention inwards may be the worst service we can do him.[1]

It is a singular fact that while private persons have an almost limitless faith in the power and wisdom of medical men, believing often enough that the doctor can read the inmost secrets of their bodies without asking a single question, yet there is among the general public a rising jealousy and

[1] I was sent for on one occasion to see a young married lady who had settled in Weston. She informed me that she was under the care of a London consulting physician, to whom she went every fortnight to report herself and receive further advice, but that she felt that she would be safer if a local medical man were thoroughly acquainted with her case and could be called in on emergencies. The London doctor had written out a diary for her use, and she was endeavouring to observe its directions with religious fidelity. It began as follows: "8 a.m.—rise; 9—breakfast, cup of freshly-infused tea with thin dry toast, and lightly-boiled egg or small piece of white fish; 9.30—bowels move; 10—go out for half-an-hour's quick walk." And so on, the whole day being mapped out in alternate meals, walks, drives, and domestic duties, as if the patient were some kind of brainless mechanism. Her efforts to follow with literal strictness the "doctor's orders" were pathetic. You will hardly believe me when I assure you that there was nothing wrong with that lady except some very slight symptoms of dyspepsia, but she was being fast moulded into a confirmed chronic invalid. My advice to her was to put the diary in the fire, and use her own common-sense in arranging her meals and forms of exercise; and I am glad to say that she had the wisdom to follow this advice, and in a week or two was laughing at her own folly and enjoying perfect health.

A wealthy bachelor, living in a hotel, once sent for me. I asked what he fear of the profession. They recognise that the profession is becoming possessed of sanitary knowledge of truly national importance, and is desirous of pressing on all public authorities and managers of institutions an enlightened obedience to the laws of health. It is largely to this that the improved condition of our unions, reformatories, schools, &c., is due. But the public do not approve of this pressure. They object to being controlled even by the highest medical opinion, and are afraid of the rise of a medical priesthood claiming infallible rights of sanitary compulsion. Parliament, which best reflects public sentiment, has just afforded us an instance of this in the Vaccination Act of the present year, wherein the rights of conscience have received a new kind of extension, being made legally available to secure exemption from what virtually the whole medical world regards as a national hygienic necessity. Far be it from me to claim an unerring judgment for our profession. Let us remember that the Infectious Disease (Notification) Act, now admitted to be of valuable public service, was at first opposed by many of us as involving a breach of professional secrecy, an objection now regarded as a mere shadow.

What is wanted is a more hearty co-operation and mutual complained of. "Oh, *you* must tell me that," he replied. I said he must name his symptoms, what did he *feel?* "Oh, I sent for you to tell me that. Would you like to examine me?" I told him if he wished it I should be pleased to do so. He accordingly stripped and lay down on a sofa. "What do you find?" he asked. "All your organs are in their right places and of normal size," I answered. "You don't find the stomach dilated?" To my negative reply he exclaimed triumphantly, "Then you differ from Sir X. X.," mentioning an eminent physician; "he told me my stomach was dilated." "How long ago?" I asked. "Three months." "And you have been under his treatment ever since?" "Yes." "Well," I said, "be thankful; the treatment has been quite successful, and you are now well!" I found this gentleman weighing with scales every atom of food which he swallowed, and living a slave to his stomach. One day he stopped me when out driving and asked me to furnish him with a list of the proteids and carbohydrates, with their exact quantities, which a man of his age and weight should consume in twenty-four hours, and was both disappointed and indignant when I counselled him to leave his stomach alone, to eat when hungry and leave off when satisfied, and to occupy himself with other and outside interests.

These may be absurd instances, but similar if less extreme ones are only too common.

understanding between the profession and the public, and especially the education of the latter in the real value of the doctor's work. So long as the medieval conception of disease as an entity, to be exorcised by some patent "cure" or by some rare and mysteriously gifted individual, holds the field, so long must the legitimate members of the profession expect hindrances in the execution of their work. What vexatious opposition we meet with in seeking to hold *post-mortem* examinations! What time and argument are wasted in the vain task of persuasion, and how often it happens that a blank negative is our fate in the very cases that we have an almost passionate desire to clear up!

We have, of course, to deal with a very wide-spread sentiment against the supposed mutilation of the dead, and a certain class generally attribute the doctor's wish for a necropsy to idle curiosity, or regard it as a confession of incompetence in diagnosis. At St. Thomas's, Guy's, and elsewhere a clear statement of the rules of the hospital is hung up in the entrance hall, including one that the Governors reserve to themselves, in the interests of the public and as one of the conditions of admission, the right to have a *post-mortem* examination performed on every person dying in the hospital. It is further mentioned in the same statement that if any relatives desire to object to the performance of a *post-mortem* they shall state this objection to the steward, and if after consultation with the medical officer he is of opinion that there is no urgent need for a necropsy, he is authorised to dispense with it. Under this plan the steward of St. Thomas's informs me that very few objectors are met with. I do not know the rules of the Bristol Royal Infirmary or of the Bristol General Hospital, but what seems to me most necessary is that in *all* hospitals throughout the country *post-mortem* examinations should be a matter of course, and that the public mind should become accustomed to this on finding that it is the universal custom.

The public sadly want education on these and cognate matters connected with the profession; but we may hope for the growth in time of the sense that what raises the standard, moral and scientific, of the latter is distinctly beneficial to the former. The first step towards forming that conviction must be the teaching of elementary physiology in every school in the land, with the corollary that the symptoms of disease are the expressions of deranged physiological processes. The abysmal ignorance of physiology met with now and again even among apparently educated people is astonishing, and diminishes our surprise at the strange conceptions of disease and its cure which many persons hold.

There must be a crusade of intelligence against the lies of the advertising quack. It is a curious phenomenon that some minds always favour the illegitimate in medicine, and that among the most zealous patrons of the quack fraternity are always to be found a number of "ladies of quality."[1] The "Mattei Home," in London, has an impressive list of titled ladies decorating its annual report as patrons and believers. A further outcome of improved education and intelligence, it is to be hoped, will be a Parliamentary statute making unqualified practice illegal. The General Medical Council should be empowered not only to remove names from the Register, but to deprive a man guilty of infamous conduct of his license to practise as well, whether for a time or in permanence. The Council should have power to proceed against every one who

[1] These are typified in Lady Blanche Fitzague, one of the guests at the dinner party at "The Evergreens," described by Thackeray in his inimitable *Book of Snobs*: "I am inclined to believe [her ladyship] had a wet *compresse* around her body, on the occasion when I had the happiness of meeting her. She doctors everybody in the neighbourhood, of which she is the ornament; and has tried everything on her own person. She went into Court, and testified publicly her faith in St. John Long: she swore by Dr. Buchan, she took quantities of Gambouge's Universal Medicine, and whole boxfuls of Parr's Life Pills. She has cured a multiplicity of headaches by Squinstone's Eye-snuff; she wears a picture of Hahnemann in her bracelet and a lock of Priessnitz's hair in a brooch. She talked about her own complaints and those of her *confidante* for the time being, to every lady in the room successively, from our hostess down to Miss Wirt [the governess], taking them into corners, and whispering about bronchitis, hepatitis, St. Vitus, neuralgia, cephalalgia, and so forth. I observed poor fat Lady Hawbuck in a dreadful alarm after some communication regarding the state of her daughter Miss Lucy Hawbuck's health, and Mrs. Sago turn quite yellow, and put down her third glass of Madeira, at a warning glance from Lady Blanche."

Equally amusing references to the aristocratic medical lady might be quoted from George Eliot and other writers.

practises without a license, and thus put a stop to the evil deeds of bone-setters and other quacks.[1]

I have already referred to the competition now existing in the profession. A most serious result of this, and one which of late has received much prominence in the medical journals, is the sweating of doctors by so-called "Medical Aid Associations," which are managed by laymen and run for their profit, which make way by touting and canvassing even among well-to-do people, and which place the doctor in a position of complete servitude. In South Manchester it was found that one of these associations had distributed its circulars to occupiers paying a yearly rent of £40 and £50. Now, it is true that the working-classes generally cannot afford the ordinary fees due to medical men; but the provident or club system, by which members pay a small regular contribution both in health and sickness, is well adapted to meet this difficulty. The Registered Friendly Societies of the United Kingdom, which provide medical attendance for their members on this system, constitute together a very numerous and powerful organisation.

[1] In an excellent letter in a recent number of the *Westminster Gazette*, signed "Medicus," on "What is a quack?" I find these words, which I heartily endorse: "The quack who ought to be put down by legislation is the ignoramus who is unacquainted with the elementary laws of anatomy, physiology, pathology, &c. . . . That doctors differ among themselves only shows the difficulty and intricacy of their work, and is a strong argument against allowing totally ignorant persons to attempt it. The fact that two cabmen take a different view of the shortest way to get from one point of London to another is no argument for permitting men totally ignorant of the London streets, and unacquainted with the elements of driving, to ply for hire. You speak of the difficulty of the law intervening, but if the law can prevent omnibuses and cabs from plying without a license, and exercise a similar control over public-houses, theatres, &c., surely it should intervene to prevent a man from practising as a doctor without the necessary legal qualification . . . The law at present has a clear definition of a qualified practitioner, namely, one who has studied for a given number of years at a properly recognised medical school, and has passed certain recognised examinations. Such properly qualified men (and women) are put on the 'Medical Register,' which contains some 30,000 names. No one but those on this register can sign a medical certificate of the cause of death, or hold any medical post under Government, or in the Army or Navy; and I certainly can see no valid reason why the law should not go one step further, and refuse to permit any but these to practise the responsible arts of medicine and surgery at all."

Exclusive of Collecting Burial Societies, there are in the United Kingdom 23,998 friendly societies and branches with a membership of 4,203,601, and funds amounting to £22,695,039. A great many of these provide medical attendance at a contract price ranging from 2s. 6d. to 4s. or 5s. per member per annum. What is becoming a frequent practice now is for all the clubs in a town to join in providing a staff of doctors for their members, either on a like basis of contract or as salaried officers. Now, so long as these provident benefits are strictly limited to the working-class, and fair terms of agreement are arrived at between the medical man and the club for which he acts, no one need take exception to the system. Unlike legal or any other kind of advice, medical advice is a *necessity*, recognised as such by the State. It is also a necessity that the advice be *good*, and that it represent the product of expert and carefully tested knowledge and skill. But if for a mere livelihood medical men are obliged to see far more cases than they can do real justice to, they must become the merest automatons, and they degrade the status of their profession. Readers of the *British Medical Journal* have for several years past noticed a frequent editorial paragraph entitled "The Battle of the Clubs," from which it is quite clear that the tendency of these is more and more to reduce the doctor to a position of dependence and servitude, and to lower his remuneration and increase his work by admitting women and children—whose illnesses are much more frequent than those of men—at quite insufficient rates. Thus the question of professional status is coming to an issue throughout the country. At Thorne, in Yorkshire, all the medical men refused to act longer for friendly societies unless membership were confined to working-men. The clubs advertised, but without success, and the two largest conceded the demands of the medical men. Unhappily, in other places doctors have been found so wanting in loyalty as to accept the humiliating terms offered and to undersell the whole medical community of a district. The Medical Aid Associations are the worst offenders, and the abuses connected with them are quite as flagrant as those notoriously associated with our hospitals and medical charities.

There is one way to antagonise them, and it has been successfully carried out in many towns, including Weston-super-Mare. It is for the local medical men themselves to form a Provident Medical Association on these lines: (i.) the management to be entirely in their own hands; (ii.) the imposition of a wage-limit to membership; (iii.) the total avoidance of canvassing; and (iv.) the division of the profits among the staff according to the list of patients belonging to each. The Weston-super-Mare Provident Medical Association, which came into working existence about a year and a half ago, was formed with the definite purpose of bringing to nought an unscrupulous Medical Aid Association, which had established itself in the town, and at the same time of placing medical relief on a provident but really effective basis. It would in all probability have at once attained large dimensions were it not that a Provident Dispensary, worked in conjunction with the Hospital, and with a specially appointed medical officer residing there and visiting the patients in their homes, has already been in operation since the summer of 1886. That Provident Dispensary has a membership of 2,330 (adults 1,033, children 1,297), and its medical officer paid 8,179 home visits during 1897, about an equal number having attended at the hospital for treatment. The Provident Dispensary is thus a powerful rival to the Provident Medical Association. The latter, however, offers the advantage of a staff of *sixteen* medical men, from whom patients may choose their own attendant, and eight chemists, who receive 1/- a year from each patient on their list. The minimum fee paid by members is one penny per week, and in special cases this is increased. Though the membership is still small, it shows a gradual and steady increase, as will be seen from the numbers on the books at each quarter from the commencement:

| | | | |
|---|---|---|---|
| 1st quarter | ... 275 | 4th quarter | ... 400 |
| 2nd ,, | ... 315 | 5th ,, | ... 418 |
| 3rd ,, | ... 341 | 6th ,, | ... 455 |

At present it is about 470. I am glad to say that one of the results has been successfully accomplished. The Medical Aid Association has had to retire discomfited to well-merited oblivion.

If the abuses which threaten the independence, the dignity, and the efficiency of the profession are to be combated, it must be by a greatly increased sense of corporate unity in the profession, by a new *esprit de corps*, by higher ideals of conduct and honourable dealing, and by definite pronouncements by the authoritative voices of the profession in condemnation of those who turn the noblest of professions into a trade, and are ready to secure practice by underselling their brother practitioners. I repeat that much can be done by teachers in our medical schools to raise the standard of conduct, and everything which brings members of our great fraternity together in kindly intercourse and mutual understanding will indirectly serve the same end.

---

